# Human–Wildlife Conflicts in Krakow City, Southern Poland

**DOI:** 10.3390/ani10061014

**Published:** 2020-06-10

**Authors:** Sayantani M. Basak, Izabela A. Wierzbowska, Agnieszka Gajda, Marcin Czarnoleski, Maciej Lesiak, Elzbieta Widera

**Affiliations:** 1 Institute of Environmental Sciences, Faculty of Biology, Jagiellonian University, Gronostajowa 7, 30-387 Krakow, Poland; sayantani.basak@doctoral.uj.edu.pl (S.M.B.); marcin.czarnoleski@uj.edu.pl (M.C.); e.widera@uj.edu.pl (E.W.); 2 Institute of Urban and Regional Development, Targowa 45, 03-728 Warsaw, Poland; agajda@irmir.pl; 3 Maciej Lesiak, “KABAN” Maciej Lesiak, os. Albertyńskie 1-2/125, 31-851 Krakow, Poland; biurokaban@o2.pl

**Keywords:** hot spots, red fox, roe deer, wild boar, Moran’s Index, urban environments, GIS, animal-vehicle collisions (AVC)

## Abstract

**Simple Summary:**

Human conflicts with wildlife (HWC) are gradually increasing in urban areas with changing patterns of land-use and fragmentation. We investigated the occurrence of human-wildlife conflicts in Krakow city, southern Poland, based on recorded conflict incidents with wild animals in three categories, i.e., animal-vehicle collisions, intrusion to property, and damages. The three most common conflict species were red fox, roe deer and wild boar. We analysed the spatial and temporal distribution of the HWC. The results of the study can be used to identify locations of potential HWC hot spots in the city and provide information for mitigation actions.

**Abstract:**

Efforts to reduce human-wildlife-conflict are integral to wildlife management and conservation in urban habitats. In our study, we identified the HWC situations in urban areas of Krakow city, based on animal-vehicle collisions, intrusion to property, and damages. Hot spot analysis and Moran’s Index were used to identify the location of maximum potential conflict. We analysed 2512 incidents in which animals (of which 85% included mammals and 15% birds) were involved in conflict situations between 2007 and 2013. A significant seasonal variation was observed among the animals. We also identified roe deer (50.23%), red fox (22.80%) and wild boar (11.40%), as the three prominent conflicted animals. Getis–Ord Gi* analysis was used to identify spatial clusters of conflict. A significant spatial association was found in the location of clusters of hot spots in specific land-use based on Moran’s Index. Hot spots of roe deer and wild boar were high in grasslands and in forest and for red fox in built-up area. The results underscore the notion that conservation and wildlife management efforts must take into account differences in the seasonality of HWC among species. This information can be used to inform mitigation strategies.

## 1. Introduction

Human interactions with wildlife are an important part of human existence that undergo constant changes in type and intensity through time and space, and from human perspective varying on a continuous scale from positive to negative experiences. Human-wildlife conflicts (hereafter HWC) intensify when there is an overlap between demands on the land of both sides [1,2,3]. The factors influencing these interactions are diverse and sometimes distinct portraying the intricacy in wildlife and human behaviour [4,5]. The complexity of HWC rises with a gradual increase in urbanisation and reduction in green areas that are potential wildlife habitats [5,6].

Urban transitions are a global phenomenon. Combined with a large-scale of mega-urban regions and the expanding geographic urban processes, they signal a significant break in human–environmental interactions [7]. Currently, 55% of the global population reside in an urban setting [8] and this is expected to rise to 68% by 2030 [9]. Continuous human population growth and demands for new resources lead to destruction and transformation of natural habitats which consequently negatively affects various ecological aspects of ecosystem complexes and provided services [10,11,12].

The unprecedented expansion of urban areas will undoubtedly continue to transform the environments of the world, with profound consequences for biodiversity [13] in all ecosystems worldwide [14]. Even though urbanisation leads to a decrease in species diversity and causes its homogenization, there is evidence that the matrix of urban ecosystems can offer valuable habitats for several wildlife species that show plasticity and adaptability towards specific urban and suburban conditions [15,16,17].

Wildlife has existed in urban areas for as long as humans lived in settlements [6] and has already interacted frequently with humans [18]. Thus, the city would no longer exist solely to meet the demands of humans but also to serve as home to a largely abundant native flora and fauna, highlighting the need for finding ways for people and wildlife to coexist [19]. Thus, how wildlife species use urban areas, and how they utilise the available resources, has a profound impact on human-wildlife interactions [6]. Animals respond to environmental change either through dispersal, phenotypic plasticity, or through evolutionary adaptation [20,21,22].

Numerous species exhibit behavioural changes in response to altered habitats by humans by creating opportunities for their survival [23]. In many cases, wildlife co-exists in environments near humans [24]. For example, several medium-sized carnivore species such as red fox (*Vulpes vulpes*), stone marten (*Martes foina*), European badger (*Meles meles*), or coyote (*Canis latrans*) can utilise resources and shelters provided by urban habitats by decreasing their home ranges and feeding on anthropogenic food such as leftovers and trash [25,26,27,28]. Additionally, the development of nocturnal behaviour is a common form of complete human avoidance [29].

Consequently, urban ecosystems allow certain animal species such as cosmopolitan bird city dwellers like feral pigeon (*Columba livia domestica*), rook (*Corvus frugilegus*), jackdaw (*Corvus monedula*), blackbird (*Turdus merula*), house sparrow (*Passer domesticus*) or European starling (*Sturnus vulgaris*) to raise their densities compared to their presence in natural environments [30,31]. The habituation of wildlife to humans and urban environments leads to a growing number of wild animals, that show little or no fear of humans, which consequently increases HWC [32,33,34,35]. Moreover, taking into consideration the rapid human population growth rate and increasing demand for access to land and resources, it can be expected that HWC will increase in numbers even more [1,36]. Recently, the encroachment of large-bodied animals like big cats (leopard *Panthera pardus*) [37] bears (e.g., black bear *Ursus americanus*) [38] or ungulates including moose (*Alces alces*) [39,40], red deer (*Cervus elaphus*), and wild boar (*Sus scrofa*) [41] into urban lands, have arisen.

HWC is often localized in specific spatial-temporal conditions [4]. Yet, still little is known about how these interactions happen in urban habitats. Places of higher than expected occurrence of wildlife collisions along roads are called hot spots or, blackspots [42,43]. The combination of physical factors such as habitat type, terrain, the landscape is associated with the distribution of such hot spots [44]. Mapping of specific species for the localisation of hot spots is therefore needed to identify relevant conflicts to maximize efforts in indicating places with a high probability of human-wildlife conflict [45]. For this, a spatial approach identifying high-priority conflict hot spots is widely adopted [46] and appreciated by policy makers.

A greater understanding is a prerequisite for developing mitigation procedures and management strategies of urban wildlife populations [47]. Thus, the main purpose of our work was to evaluate long term data of HWC in a large metropolis to indicate land features related to a higher probability of occurrence of such incidents.

We aimed to describe conflict animal species and model spatial-temporal factors which might affect specific cases. For temporal patterns, we expected that the frequency of conflicts with mammals and birds will depend on season [17,31,48,49]. In particular, we hypothesised that the majority of conflicts for red fox will be observed in summer during dispersal of subadults, for roe deer, during establishment of the territories by males in spring and breeding season in summer, for wild boar, during dispersal of subadults in autumn [48,50]. For avifauna, we hypothesised that conflict situations would arise more frequently during seasonal migrations, mainly in spring and late autumn [17,31,51]. For spatial patterns, we generally predicted that the frequency of HWC in an urban matrix will concentrate mainly in the vicinity of green patches, i.e., forest, grassland, which are most likely to be utilised as urban habitats by wildlife [41,52].

## 2. Materials and Methods

### 2.1. Study Area

**Figure 1:**
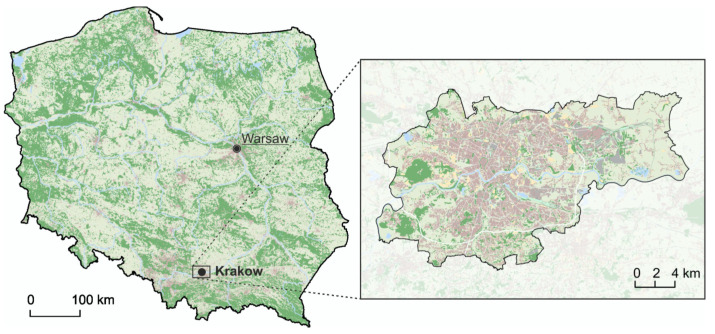
Location of the study area where human-wildlife conflicts (HWC) were recorded between 2017 and 2013.

The study was carried out in Krakow (50°03′4″ N, 19°56′1″ E), the second largest city in Poland, with a population of 775,000 in 2019 [53]. From west to east the city is crossed by the Vistula River, the largest Polish river, and its tributaries [54] (Figure 1).

Krakow is featured by differentiated land cover comprised of urban lands, agricultural areas, green patches (mainly parks, orchards, meadows, and woodlots), and watercourses, encompassing 42.6%, 46.4%, 9.3%, and 1.7%, respectively [55,56]. Forests and shrubs comprise 11% of the vegetation [57]. The city has a dense road network (3.86 km of public roads/km²). With its long history of trade and transit, transportation has always played an important role in the city. Fauna of Krakow is diverse and rich in species with recorded 75 invertebrates, 12 amphibians, 5 reptiles, 226 birds, and 42 mammal species [58]. The city is occupied by wild boar, roe deer and medium sized carnivores such as red fox and stone marten. Since the 2000s, there has been a constant increase in sightings in the area for all of these species [27,59,60]. All of them are game species that are being hunted in two hunting grounds located in the northern and south-western parts of the city. In 2013, the estimated numbers of roe deer, wild boar, red fox and stone marten in these hunting grounds were 410, 100, 145 and 52, respectively, and they increased almost three-fold by 2016–2017 [61].

### 2.2. Data Collection

HWC incidents, with a total number of 2512, in Krakow were recorded between September 2007 and December 2013 by KABAN Co., managed by Maciej Lesiak, working for Krakow Municipality and the Regional Directorate of Nature Protection in Krakow. According to the agreement between the parties, KABAN Co. was obliged to manage each HWC incident reported by municipal institutions, such as Krakow Municipality, Police, City Guard Service, Department of Environment, District Centre of Crisis Management, Krakow Animal Shelter, and Regional Directorate of Nature Protection in Krakow. The qualified KABAN Co. telephone central officers received phone calls from the above-mentioned institutions about HWC in Krakow and verified each incident by further interview followed by field intervention if required. The field intervention included HWC locality checking and monitoring, trapping HWC animals, their translocation, animal-vehicle collisions (hereafter AVC) and interventions. The details of each HWC case, i.e., date and place of an incident, animal characteristics, type of intervention, were recorded to form a database.

The HWC data were grouped into conflict categories, i.e., AVC, property intrusion, damage caused by animals in the buildings, cars or, other properties. The animals involved in HWC were classified into two taxonomic groups: mammals and birds.

### 2.3. Data Analysis

#### 2.3.1. Temporal Pattern

The relationship between seasons and HWC in Krakow city was examined. The seasons in Poland are categorised as spring (March–May), summer (June–August), autumn (September–November) and winter (December–February). We explored the pattern of seasonal variation using non-metric multidimensional scaling (NMDS) analysis which was used to produce a two-dimensional graphical representation. NMDS uses an iterative algorithm to reduce multidimensional similarity data that indicates the similarity of samples [62]. Additionally, we used constrained canonical analysis (CCA) that facilitates the study of linear interrelationships between two sets of variables. CCA develops a canonical function that maximizes the correlation between any two variables [63]. CCA allows uncovering patterns that are sometimes difficult to interpret in an unconstrained NMDS ordination [62]. In our study, we tried to maximise the correlation between season and year using HWC of animals as a response. Limited number of predictors (year) allowed better representation of the constrained ordination [64]. A linear model was used for HWC of all animals considering season as a predictor. The pairwise comparison between season was conducted by Tukey HSD adjusting p-value by the Benjamini-Hochberg procedure [65,66]. Similarly, we have estimated the seasonal variation of HWC by birds and mammals. A heat map was created to show the abundance of HWC. The statistical analyses were performed in R (version 3.6.1) vegan package [67].

#### 2.3.2. Spatial Pattern

Every HWC record was associated with the address point. The database was geocoded based on the street/number addresses and later transformed to the shapefile point file in QGIS 2.14. [68].

Hot spots of HWC were performed only for the highest abundance of such cases, i.e., roe deer, wild boar, and red fox which were the most common wildlife species involved in conflict situations in the study area. Each conflict incident was assumed to occur within the home range of an animal. For simplicity, we assumed that the individuals involved in all conflict situations were characterised by home ranges typical for the species in urban or peri-urban habitats. We use home ranges sizes described in the literature: for wild boar [59] (3 km^2^), red fox [27] (2.6 km^2^), and roe deer [69] (0.3 km^2^).

The location of each conflict situation was used as a centre point of a circle that defined the home range. The home ranges helped us to associate each conflict situation with characteristics of the urban environment–The percentage of the area covered by different types of land-use, namely build-up areas, grasslands and forest. To identify land-use types in the study area we used the National Database of Topographic Objects [70].

Spatial distribution of HWC for red fox, roe deer and wild boar was modelled. We expected that the spatial distribution of conflict situations deviates from random, showing a strong positive spatial autocorrelation. We measured this autocorrelation with a help of the Moran’s test [71] followed by hot spot analysis Getis-Ord Gi* in ArcGIS 10.3.1 [72,73], to identify areas that were either the most or the least likely to be involved in the conflict situations. This method identifies locations with either unusually high (hot spots) or low (cold spots) frequency of studied incidences, which cannot be explained by the random spatial distribution of cases, assuming at least a 95% confidence level.

Both analyses were calculated separately for each species. In this analysis, we used a hexagonal grid, which is a grid type that increases connectivity between grid units and fits better the shape of the study area [74]. Hexagons with 150-m side length were generated in ESRI ArcMap 10.3.1, and they covered the whole study area (6257 units in total). Each hexagon was characterised by information added to an attribute table such as the number of HWC and share of basic land-use types in each hexagon, i.e., forested, build up and grassland areas. We also used the hot spot analysis to identify regions with an unusually high or low occurrence of build-up areas, grasslands and forests. Ultimately, we integrated the results of hot spot analysis on conflicts and land-use, by superimposing the spatial locations of conflict situations and land-use types.

## 3. Results

### 3.1. Descriptive Statistics

Amongst 2512 of total HWC, mammals and birds made 85% (n = 2140) and 15% (n = 372), respectively. Overall, the most common type of the HWC incidents was AVC (2083 cases), followed by intrusion to property (777 cases) and damage (24 cases). Mammals contributed to HWC mainly in AVC and intrusion to property. The most HWC mammals were roe deer (50%) followed by red fox (23%) and wild boar (11%) (Supplementary Table S1). In birds, the most common incidents were AVC with feral pigeons, mute swan (*Cygnus olor*), and mallard (*Anas platyrhynchos*), which comprised 43%, 21%, and 19% of HWC, respectively (Supplementary Table S2).

**Figure 2:**
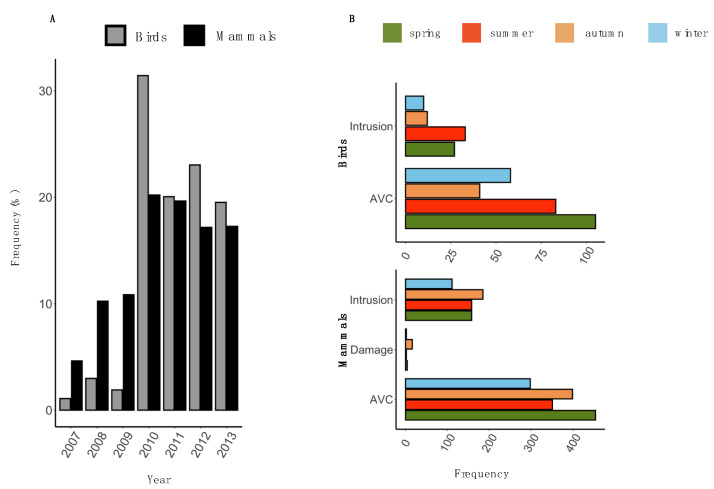
The occurrence of human-wildlife conflicts (HWC) in Krakow city between September 2007 and December 2013. (**A**) The annual frequency (percentage) of HWC incidents for birds and mammals. (**B**) The seasonal variation of the number of intrusions, damage and animal-vehicle collision (AVC) incidents among birds and mammals.

The mean frequency (±SD) of HWC per month was 4.5 ± 3.7 for all birds and 27.83 ± 7.15 for all mammals. The proportions of HWC per year showed an increasing trend for both mammal and bird species over the years with a peak in 2010 (Supplementary Tables S3 and S4). Since 2010, the frequency of HWC incidents (per month) was higher for birds than with mammals (Figure 2A).

The annual mean (±SD) number of cases of AVC, intrusion and damage cases were 214.71 ± 139.52, 87.57 ± 45.47, and 3.43 ± 5.80, respectively for mammals (Supplementary Table S3) whereas for birds the annual mean (±SD) number of cases of AVC and intrusion to property for birds was 41.42 ± 40.37 and 11.71 ± 7.57 ((Supplementary Table S4).

During nearly 6 years of the investigation, there were no damage cases recorded for birds (Figure 2B), with the highest incidents in vehicle collisions (n = 290) followed by intrusion to property (n = 82). For birds, the highest number of AVC incidents occurred in spring followed by autumn and winter, while intrusion to property was highest in autumn followed by spring and summer (Figure 2B).

Mammal-vehicle collisions (n = 1503) were the most common HWC incidents in Krakow city appearing regularly in all seasons followed by intrusion to a property (n = 613) and damage (n = 24) (Supplementary Table S4). AVC for mammals was highest during spring closely followed by summer and autumn. Frequency of intrusion cases to property was the same during autumn and spring with a little increase in summer (Figure 2B). There were very few cases of damage to property occurring mainly in summer.

### 3.2. Temporal Pattern of HWC

To identify the pattern of similarity of HWC among seasons, the NMDS graph was plotted (Supplementary Figure S1). The graph showed a significant variation among seasons in the first axis (47.58%) and years in the second axis (26.82%).

The first axis variation indicated a cluster of seasons between winter and spring and between summer and autumn with a variation of 47.58%. Supplementary Figure S1 showed the seasonal pattern of all HWC.

CCA was performed to explore the occurrence of HWC in different seasons and different years. The results revealed (Supplementary Figure S1) a strong clustering by seasons. The explained variation of the model shows 57.38% in component 1 and 30.65% in component 2. As shown in Supplementary Figure S1, the first two CCA axes represented over 88% of the variance. In the first axis, HWC in summer and autumn showed a strong positive correlation with seasons in the years 2011, 2012 and 2013; conversely, winter and spring correlated negatively with the years. On the second axis, we found positive correlations for winter and autumn and a strong negative clustering for spring. CCA plot suggested that there might be a strong influence of seasons on the incidences of HWC in each year. The explained variance of the model among the seasons was 25.99% which was statistically significant upon PERMANOVA (constrained: season = 0.1759; unconstrained = 0.2656; *p* value = 0.01) (Supplementary Table S6).

**Figure 3:**
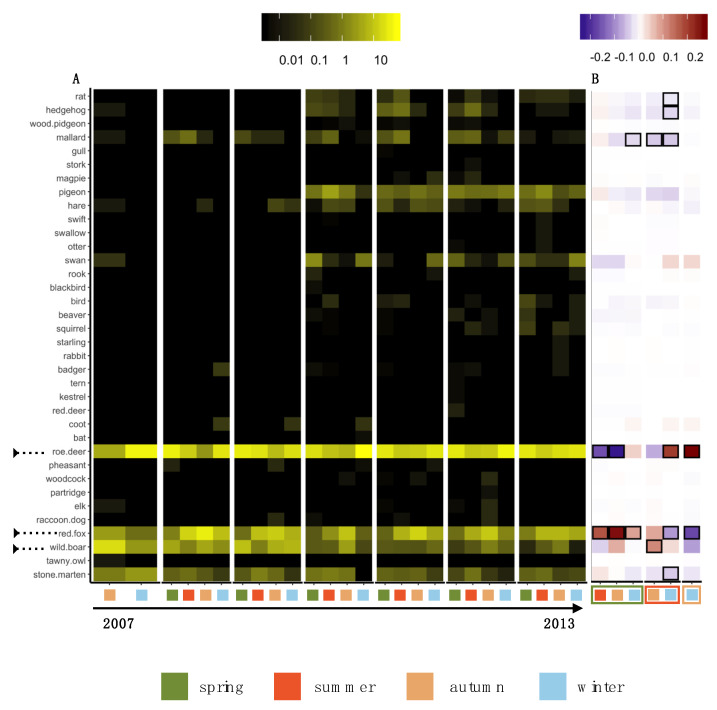
Seasonal diversity of human-wildlife conflicts (HWC). (**A**) Heat map showing the abundance of HWC in percentage for 2007–2013 for each season for all animals. The marked arrows on the left represent the animals with the most occurrence of HWC. (**B**) Pairwise seasonal effect using generalised linear model and Tukey HSD for computing significance (*p* < 0.05). The colours represent the mean difference of HWC between seasons. Significant differences (*p* < 0.05) are marked by framed boxes.

The heat map of HWC for all animals across seasons in different years showed that roe deer, red fox and wild boar were the most abundant species, appearing in all seasons throughout the years (Figure 3A).

Upon pairwise statistical comparison, a seasonal variation was observed for the animals without the effect of years (Figure 3B). For roe deer, the significant mean difference for autumn in comparison to spring was MD = −0.2294 indicating higher incidences of HWC in spring than in autumn (*p* = 0.0025). Similarly, the significant mean difference was higher for winter in comparison to autumn (MD = 0.27) and summer (MD = 0.20) indicating high HWC in the winter season (*p* < 0.01) (Supplementary Table S5).

**Figure 4:**
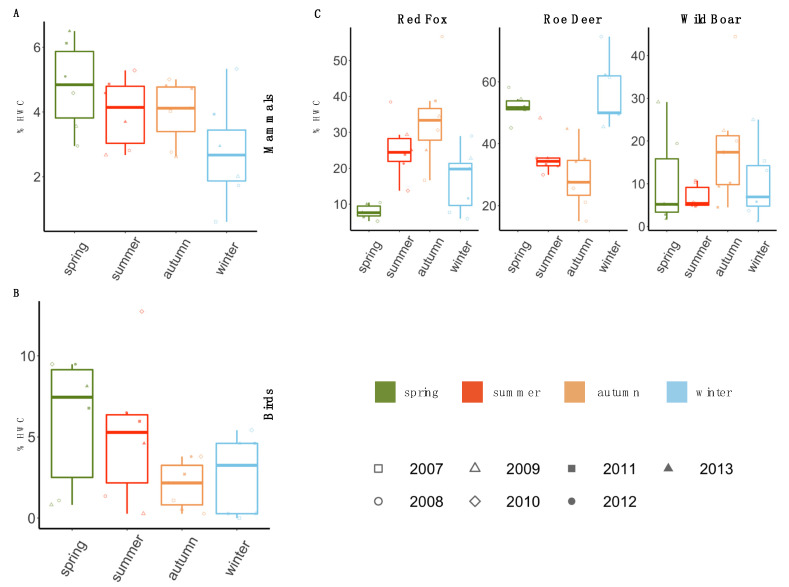
Box plot showing a seasonal variation of human-wildlife conflicts (HWC) for mammals and birds. Box: middle 50% of the data (25th–75th percentile); horizontal line: median; dots: outliers (**A**) shows overall seasonal variation for mammals. (**B**) shows overall seasonal variation for birds computed using the linear model considering year as a random factor. (**C**) depicts a seasonal variation of red fox, roe deer and wild boar. The colours represent the seasons and the shapes represent corresponding years.

The distribution of the total percentage of HWC for mammals (Figure 4A) showed variation among seasons with the highest number of cases observed in spring followed by summer and autumn. The lowest number of cases of conflicts was detected in the winter season.

High percentage of cases of conflicts was noticed in spring for all birds (Figure 4B). However, birds showed the least HWC in autumn followed by winter.

The three mammal species that caused most conflicts (red fox, roe deer and wild boar) were separately analysed (Figure 4C). While red fox and wild boar showed the highest frequency of conflicts in autumn, roe deer, on the other hand, showed the least percentage of HWC in autumn. Spring season was characterised by the least incidences of HWC for both red fox and wild boar, on the contrary, it was quite high for roe deer (Figure 4C).

For red fox, the observed pattern was the opposite. The occurrence of HWC was higher in autumn with a significant mean difference between autumn and spring MD = 0.2574 (*p* < 0.0001) and between winter and autumn MD = −0.1696 (Supplementary Table S5). Wild boar exhibited more conflict incidences in autumn compared to summer MD = 0.1140 (*p* = 0.01) (Figure 4B).

In conclusion, red fox and wild boar, in general, appeared to have the highest frequency of HWC in the autumn season, in contrast to roe deer which exhibited its higher incidences in spring and winter.

### 3.3. Spatial Pattern of HWC

**Figure 5:**
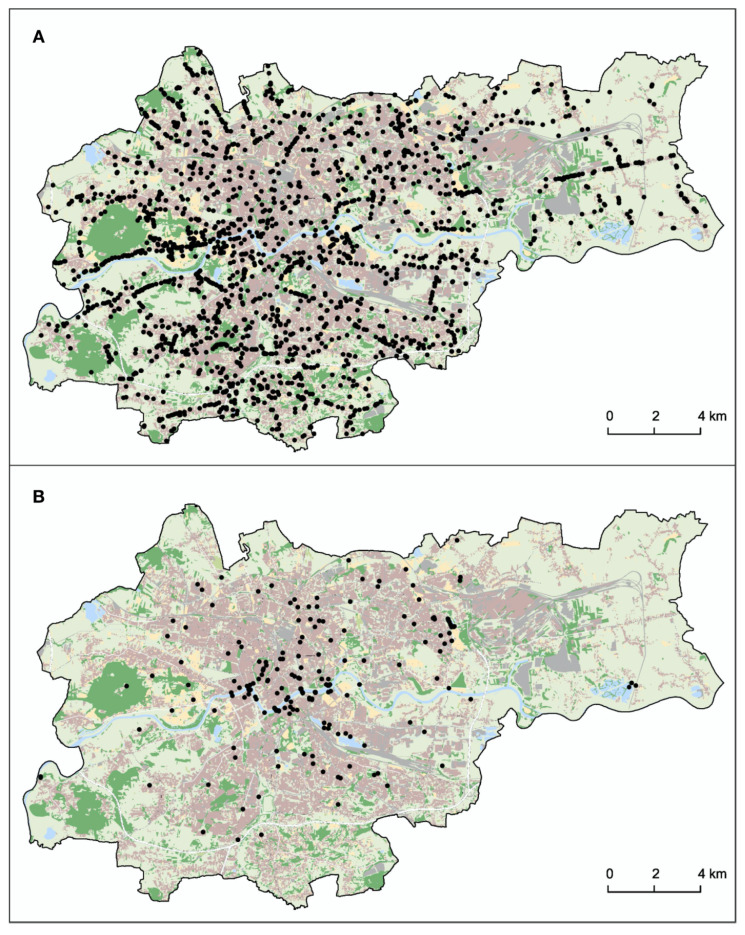
Human-wildlife conflicts (HWC) in Krakow between 2007 and 2013. The black dots represent specific locations. (**A**) shows the distribution of HWC for mammals (**B**) shows the distribution of HWC for birds.

A spatial representation of HWC in Krakow city determined the location of these conflict situations (Figure 5). Overall the distribution was spread throughout within the city boundaries.

In our study, the values of Moran’s Index (Table 1) showed significant results with *p*-value < 0.0001. Consequently, we rejected the null hypothesis that distribution of HWC for all the three mammals was random. This indicates some form of clustering in the occurrence of conflicts. A z-score between −1.96 and +1.96 indicates random distribution in Moran’s statistics. The positive z-score values for red fox (+14.64), roe deer (+17.25) and wild boar (+24.69) indicate that the pattern of HWC is spatially clustered. This was further validated when the Moran’s Index for the three mammals was greater than 0, where 0 denotes perfect randomness [75,76].

The Moran’s Index for wild boar was almost twice (0.18) than that of the red fox (0.107) indicating that wild boar showed a more spatial clustering than red fox. The index value for roe deer was 0.12 which means the clustering of it was more than for the red fox but lower than for the wild boar.

Hot Spot Analysis was conducted to identify statistically significant hot spots and cold spots using Getis-Ord Gi* statistic for the three mammals (red fox, roe deer and wild boar) with the highest frequency of HWC. This Gi* statistic returned a z-score which is then used to determine the clustering of HWC. For statistically significant positive z-scores, the larger the z-score is, the more intense the clustering of hot spots indicating higher HWC. On the contrary, statistically significant negative z-scores, indicate more intense clustering of cold spots thereby suggesting the least conflict situation.

Spatial hot spot distribution of red fox showed small patches of conflicts that occurred in the central and eastern parts of the city (Supplementary Figure S2). The distribution for red fox showed that hot spots clustered around built-up areas, which indicated severe conflicts in this zone (with 99% confidence level). The cold spots were noticed in the eastern part of the city depicting lower HWC in these areas.

Distribution of the locations of hot spots for roe deer (Supplementary Figure S3) showed scattered clusters of HWC in the southern, western, and north-eastern area of the city. Hot spots of roe deer were majorly located in forest areas (with 99% confidence level) and grasslands. Cold spots were mainly concentrated in the eastern and north-eastern parts of the city that suggested lower chances of the conflict situation in this area.

HWC with wild boar, on the other hand, were more clustered in the western part of the city and (Supplementary Figure S4) showed high concentration of hot spots in grasslands and forests (with a 99% confidence level). The hot spots with wild boar in built-up habitats were located in the southern part of the region. Cold spots, on the other hand, were mostly in the eastern part of the city. The locations of conflicts with wild boar and roe deer largely overlapped with each other.

## 4. Discussion

Cities are complex ecosystem [77]. High densities of the human population, increased infrastructure and land transformation create highly fragmented landscapes with mosaics of various land-use. By the provision of profitable food and shelter, they create potential habitats for numerous vertebrate animals, mainly birds and mammals [17,78]. This is particularly observed in the case of avifauna, which according to several studies shows increased species richness and densities in city environments [17,79,80]. For example, it was [81] reported report that 20% out of 10,052 global bird species were recorded in the cities spread all over the world.

In our study, we recorded 372 conflict incidents with 19 bird species in Krakow city, with 78% of only bird-road collisions. The most common conflict bird species were feral pigeon, mute swan and mallard. While feral pigeons are typical urban species known as city dwellers worldwide, it is surprising to observe the other two species, which are associated with natural and wild wetlands [82]. Indeed, in Krakow, both species are observed in the river Vistula which passes through the city core. Both bird species are fed by people on riverbanks. They stay over winter, even though they are migratory [31]. Moreover, mallards were commonly found on properties, with several cases of birds making their nests on balconies in the block of flats, showing no fear towards people [83]. Anecdotic are incidents with mallard ducks and ducklings over passing streets in Krakow and causing traffic congestion.

The availability of food is a prime factor that enhances the survival of birds in the cities [31,79]. In Poland, it can be observed that in winter season, almost twice higher densities of birds are observed in urban compared to rural habitats [84]. Consequently, having higher densities, some bird species are involved in HWC, mainly road collisions [4,85].

Mute swans are large birds, with the body mass of adult females up to 11 kg and males 13 kg [86], most of the conflict incidents recorded in Krakow with this bird species was AVC having a devastating effect on vehicles involved and their drivers, fortunately non-fatal. Our record is consistent with findings by [87] who found that species with lower reproductive rates, higher mobility, and larger body sizes were more commonly involved in road collisions.

A recent study by [88] showed that higher bird congregations in the cities are associated with seasons and specific diet especially in spring and winter. In our study, the number of human-bird conflicts was more in spring and summer, which might be associated with the breeding season of birds determined by the temperate environment in Krakow area. Interestingly the number of human bird conflict incidents increased over six years of the study. However, we do not know how to explain this trend and we do not know which factors could affect the high peak of the conflicts in 2010.

We recorded over 2100 HWC with mammals in Krakow with two ungulate species, i.e., roe deer and wild boar and two meso-carnivores, i.e., red fox and stone marten. For birds, the predominant conflicts were road collisions and intrusion to property. HWC with mammals appeared more often in spring and summer compared to autumn and winter seasons. The analysis of the most numerous incidents with specific species revealed differences in the seasonal abundance of conflicts. Roe deer was more commonly involved in road collisions and intrusion to property in spring and winter. Incidents of the most common intrusion to property followed by road collisions with wild boar took place mostly in autumn. Conflict incidents with red foxes were recorded in autumn, summer and winter. These patterns of HWC are consistent with reports of AVC [41,89,90,91].

The seasonal variation of HWC with the above-mentioned species can be related to breeding seasons and dispersal of subadults. In case of roe deer, the spring season coincides with establishments of territories by males [90], parturition (April–May), and later in summer (July–August) with the rutting period [92]. While wild boar’s breeding season takes place in late autumn [93]. Red foxes’ parturition is between March and May, followed by dispersal of subadults in October and breading in January–March [91].

We were able to identify several hot and cold spots across Krakow city that represent locations with significantly high or low regions of HWC. The Moran’s Index was used to identify spatial dependence of HWC presented in a region [94]. Positive values (0.107 for red fox, 0.127 for roe deer and 0.180 for wild boar) with significant p-value (*p* < 0.0001) revealed the existence of a potential spatial pattern of distribution of the HWC.

The geographic pattern and change over different land-use types in the hot spots may reflect the wide differences in the clustering of conflict situations for specific animal species. Wild boar had an index value (0.180) greater than red fox (0.107) indicating that conflicts situation with wild boar has a more spatial clustering. This, in turn, would reflect wild boar to be concentrated in one part of the city while red fox projects a more dispersed distribution throughout the city.

We found out an association between hot spots of HWC and spatial distribution of the potential habitats. Hot spots with wild boar and roe deer were related more to grasslands and forests, while red fox built-up habitats. Again, the results are consistent with other reports. Both ungulate species are common in urban habitats in Poland [31,59,60]. Although showing high plasticity of habitats occupancy, they still require within their home ranges the presence of shrubs and forest patches, which allow for shelter and food provisioning [41,43]. Red fox as typical generalist species with an omnivorous diet easily adapts to the urban cores, being medium-size animals and with nocturnal activity can avoid confrontation with humans [27,91].

The hot spots of conflicts with red fox indicate the expansion and deepening of human footprints in the form of an increase in the complex anthropogenic landscape [44]. Our analysis highlighted the built-up area as the most conflict location for red fox while grassland and forests served a greater conflict situation for roe deer and wild boar.

As urban encroachment expands on to natural environments consequently more wild animal species are more prone to potential conflicts with humans [47,95]. Conflicts with wildlife can cause material and economic losses and may include attacks on humans, the transmission of zoonoses, damage to crops and property, predation on livestock and pets [2,3]. HWC appears in a non-random spatio-temporal cluster and is observed more commonly in transition lands in suburban habitats near the periphery of natural habitats such as forests [6,26,85].

## 5. Conclusions

Our results have two major implications for wildlife management, including conservation. Firstly, we demonstrated that the locations of ungulate hot spots of HWC were mainly clustered in green patches of the city, implying the requirement of greater focus in these places. Secondly, HWC varied significantly in different seasons indicating that the focus of future efforts should have a strong dependence on animals and their activity patterns to different seasons. We identified the most important zones (hot spots) of the city connected to the locations of the highest potential conflict risks with wild animals. Further studies should be aimed at understanding the opinion, attitudes and coordinated management of local communities, as well as the improvement of HWC in urban environments. Our results identify hot spots of HWC across the city and inform strategies for reducing conflict situations [2,96].

## Figures and Tables

**Table 1:** The summary of Moran’s autocorrelation coefficient analysis for human-wildlife conflict (HWC) locations in Krakow city for red fox, roe deer and wild boar.

Species	Moran’s Index	Expected Index	Variance	Z-Score	*p*-Value
Red fox	0.107638	−0.000160	0.000054	14.641317	0.000001
Roe deer	0.126715	−0.000160	0.000054	17.249950	0.000001
Wild boar	0.180016	−0.000160	0.000053	24.686691	0.000001

## Supplementary Materials

The following are available online at https://www.mdpi.com/2076-2615/10/6/1014/s1, Figure S1: Seasonal variation of human-wildlife conflicts (HWC) through all years of study. Figure S2: Spatial distribution of hot spots and cold spots of human-wildlife conflicts (HWC) with red fox in Krakow city shown on different land-use types. Figure S3: Spatial distribution of hot spots and cold spots of human-wildlife conflicts (HWC) with roe deer in Krakow city shown on different land-use types. Figure S4: Spatial distribution of hot spots and cold spots of human-wildlife conflicts (HWC) with wild boar in Krakow city shown on different land-use types. Table S1: Conflict incidents with mammalian species in Krakow city recorded between September 2007 and December 2013.; Table S2: Conflict incidents with avifauna in Krakow city recorded between September 2007 and December 2013.; Table S3: Yearly and monthly of conflict incidents with mammals in Krakow city recorded between September 2007 and December 2013.; Table S4: Yearly and monthly of conflict incidents with avifauna in Krakow city recorded between September 2007 and December 2013.; Table S5: Summary of pairwise linear generalised model with post hoc Tukey test (Heat map) for conflict incidents with mammals in seasonal comparison.; Table S6: Summary of constrained canonical analysis (CCA) of all HWC seasonal variation.; Table S7: Summary of pairwise linear generalised model with post hoc Tukey test (Heat map) for conflict incidents with mammals in seasonal comparison.
